# Diagnosis and Management of Autoimmune Hemolytic Anemias

**DOI:** 10.3390/jcm11206029

**Published:** 2022-10-13

**Authors:** Wilma Barcellini, Bruno Fattizzo

**Affiliations:** 1 Haematology Unit, Fondazione IRCCS Ca’ Granda Ospedale Maggiore Policlinico, Via F. Sforza 35, 20100 Milan, Italy; 2 Department of Oncology and Hemato-Oncology, Università degli Studi di Milano, Via Festa del Perdono 7, 20122 Milan, Italy

Autoimmune hemolytic anemia (AIHA) is usually categorized, as other immune-mediated cytopenias, in so-called benign hematology, and it is consequently managed in various settings, namely, internal medicine, transfusion centers, hematology and, more rarely, onco-hematology departments. The disease is highly heterogeneous; while most cases are mild/moderate and easy to diagnose and treat, an increasing proportion of patients experiences hyper-acute onset and may present to the emergency room or develop chronic behavior with multiple relapses. Moreover, AIHA may be secondary or associated with a variety of conditions such as lymphoproliferative syndromes, other autoimmune diseases, infections, immunodeficiencies, transplants, and drugs, including novel anti-cancer therapies, which may further complicate the diagnosis and management. Steroids, immunosuppressants, and splenectomy had been the sole therapies available for decades, while recently, several target therapies became available or are now under active development.

In this Special Issue, several aspects of this complex scenario are addressed, starting with pathogenesis [1], which is mainly driven by autoantibodies, but also involves other effectors, such as complement, phagocytes, lymphocytes, cytokines, as well as the important and detrimental mechanisms of ineffective marrow compensation. The review also focuses on the direct antiglobulin test or Coombs test, which is the cornerstone of the diagnosis but has several drawbacks, being neither 100% specific (it can be positive in healthy subjects without hemolysis) nor 100% sensitive (about 5–10% of AIHA cases are DAT-negative). The DAT allows an important distinction to be made between warm AIHA (wAIHA) and cold agglutinin disease (CAD). In the former, the autoantibody is an IgG that binds erythrocytes at 37 °C, may activate complement in few cases, and determines extravascular hemolysis mainly in the spleen via Fc-mediated phagocytosis. wAIHA is the most frequent form, and it can be primary or secondary to several conditions, as highlighted in the meta-analysis of a total of 4311 patients [2]. CAD is the second-most frequent AIHA form due to an IgM autoantibody that spontaneously agglutinates erythrocytes at cold temperatures and strongly activates complement. The hemolysis is mainly extravascular in the liver (via C3b-opsonized erythrocytes) or intravascular in the most severe cases. An important consequence of these different pathogenic mechanisms is that splenectomy is effective in wAIHA, whilst it is contraindicated in CAD [1]. The review mentions rituximab therapy, and this is illustrated in more detail in a dedicated manuscript [3], which describes the mechanisms of action of the drug (B-cell depletion and immunomodulatory effects on cytokines and T-regs) and several retrospective and prospective trials conducted in the last 15 years. Overall, responses are observed in 70–80% of wAIHA relapsed/refractory to steroids, with a median duration of response of 1–2 years and a median time to response of 4–6 weeks. The drug is also effective in the front-line setting, in combination with other drugs (cyclophosphamide and dexamethasone), and in forms of secondary to chronic lymphocytic leukemia (CLL), lupus erythematosus, and common variable immune deficiency. In CAD, rituximab is indicated as a first-line treatment, given the poor response to steroids, with a lower overall response rate (~50%), which is rarely complete and durable (6–11 months). The addition of bendamustine leads to increased and more persistent responses with an acceptable safety profile, while its combination with fludarabine considerably increases the infectious risk. This manuscript also addresses the variable and different doses utilized and the possible side effects, including infectious risk.

This underestimated issue is further expanded in a dedicated review [4] that provides a detailed description of infections (viral, bacterial, mycotic, and others) in various settings (steroids, splenectomy, rituximab, classic immunosuppressive agents, and new target drugs), and other diseases with an intrinsic infectious risk (primary immunodeficiencies, systemic autoimmune diseases, lymphoproliferative disorders, and solid organ and hematopoietic stem cell transplants) which may be associated with secondary AIHA. Moreover, viral and bacterial reactivations during immunosuppressive therapies are discussed, along with suggested screening and prophylactic strategies. Some particular aspects are further addressed, namely, AIHA in pediatric settings [5], in association with congenital anemias [6], in patients with liver and bowel disorders [7], and in CLL [8], with a focus on novel therapeutic agents in the latter, such as ibrutinib, idelalisib, and venetoclax, mostly in association with rituximab. The Special Issue also describes new target therapies for primary AIHA [1], either directed against antibody-producing B-lymphocytes/plasmacells (ofatumumab, alemtuzumab, and daratumumab) or phosphatidylinositol 3-kinase signaling (parsaclisib). In CAD, promising results are reported with complement inhibitors (sutimlimab and pegcetacoplan) and in wAIHA, several trials are running with inhibitors of the spleen tyrosine kinase (fostamatinib) or of the neonatal Fc receptor, with the latter being an interesting new strategy aimed at increasing the clearance of IgG autoantibodies.

AIHAs may be challenging and life threatening. This issue is addressed in several manuscripts, firstly in the review on Evans syndrome [9], a very rare condition where AIHA is concomitant or associated with immune thrombocytopenia (ITP), and/or rarely autoimmune neutropenia. The disease is frequently severe, marked by several relapses and refractoriness to therapies (which also include thrombopoietin receptor agonists for ITP), infectious and thrombotic complications, and overall reduced survival rates. Additionally, the complexity and severity of AIHA is highlighted in the manuscript describing instructive cases, such as hyper-acute AIHAs that require treatment in an Intensive Care Unit, the development/reactivation of the disease in pregnancy, and multifaceted AIHAs following hematopoietic stem cell transplant and novel anti-cancer therapies with checkpoint inhibitors (nivolumab, pembrolizumab, ipilimumab, and atezolizumab) [10]. Finally, this Special Issue includes the description of two cases of AIHA with underlying misdiagnosed diffuse large B-cell lymphoma, which rapidly evolved to hemophagocytic lymphohistocytosis (HLH), a rare and life-threatening hyperinflammatory condition that may be primary or secondary to many diseases [11]. A timely diagnosis of HLH is paramount but challenging, as it relies on non-specific clinical and laboratory criteria. The manuscript also describes the diagnostic challenges and a literature review of secondary HLH in the internal medicine setting.

In conclusion, this Special Issue encompasses the numerous and heterogeneous aspects of AIHA, starting with some elements regarding disease pathogenesis and the basic distinction between cold and warm forms that require quite different therapies. Another pivotal issue is a prompt recognition of the underlying disease, which may complicate the diagnosis and management. Additionally, several specific settings (pediatrics, pregnancy-related, hyperacute, iatrogenic AIHA, and cases complicating congenital anemias) may further complicate AIHA diagnosis and require special attention. Besides steroids and splenectomy (when indicated), rituximab is becoming a well-consolidated practice in *real life*, although it is not available/indicated worldwide. However, severe, refractory, and early-relapsed patients represent a challenge and an unmet clinical need. Luckily, in recent years, a growing number of trials have been running, which will raise the problem of how to choose the right therapy for each patient in the future. At the same time, this expanded therapeutic arsenal will increase the possibility to treat (and hopefully cure) the disease.
